# Molecular analysis of pancreatic acinar cell cystadenomas: Evidence of a non-neoplastic nature

**DOI:** 10.3892/ol.2014.2163

**Published:** 2014-05-22

**Authors:** FRANK BERGMANN, SEBASTIAN AULMANN, THILO WELSCH, ESTHER HERPEL, JENS WERNER, PETER SCHIRMACHER, HENDRIK BLÄKER

**Affiliations:** 1 Institute of Pathology, University of Heidelberg, Heidelberg D-69120, Germany; 2 Department of General Surgery, University of Heidelberg, Heidelberg D-69120, Germany; 3 Institute of Pathology, Charité Berlin, Campus Mitte, Berlin D-10117, Germany; 4 Department of Surgery, University of Dresden, Dresden D-01307, Germany

**Keywords:** pancreatic tumor, cystic lesion, clonality, molecular

## Abstract

The biology of pancreatic acinar cell cystadenomas has not been clearly defined. However, a non-neoplastic process, caused by a cell differentiation failure leading to a cystic transformation, has been discussed, as well as a benign neoplastic lesion. Pancreatic acinar cell cystadenomas usually consist of thin-walled unilocular or multilocular cysts, and mural nodules have been described in two cases of a recent series. In one of these nodules, chromosomal imbalances were detected, which provided preliminary evidence for a neoplastic process. The aim of the current study was to further characterize the lesions by molecular analyses. In four cases without mural nodules, the clonality was assessed by performing mutational analyses within the highly variable displacement-loop region of the mitochondrial DNA. As a result, no closer correlation was identified between different foci within the tumors than between the tumors and adjacent normal pancreatic acinar tissue, indicating polyclonality of these lesions. Further molecular analyses revealed no mutations of the *β-catenin* and *K-ras* genes. In addition, no immunohistochemical evidence was identified for mutations of Smad4 or p53. In conclusion, the results of the current study demonstrated that pancreatic acinar cell cystadenomas are non-neoplastic lesions, with the potential exception of those rare cases with mural nodules.

## Introduction

Cystic lesions of the pancreas comprise non-neoplastic lesions, which are frequently associated with pancreatitis, as well as a broad spectrum of benign and malignant neoplasms (1,2). Histomorphologically, the most common cystic pancreatic neoplasms are intraductal papillary mucinous neoplasms (IPMNs) or serous cystic neoplasms, whereas mucinous cystic neoplasms, solid pseudopapillary neoplasms, cystic pancreatic neuroendocrine tumors and other cystic neoplasms occur less frequently (1).

On rare occasions, cystic pancreatic tumors show acinar differentiation. In this case, acinar cell cystadenocarcinoma, a variant of acinar cell carcinoma, must be considered for differential diagnosis (3). Furthermore, 26 cases of cystic acinar tumors lacking any features of malignancy have been reported (4–11). A neoplastic and a non-neoplastic nature of these lesions has been debated and, as a working hypothesis, the evidently benign lesions have been designated as acinar cell cystadenomas (4,11). In the current study of four cases of acinar cell cystadenomas, which were investigated clinically, pathologically and by means of molecular analysis, evidence for their non-neoplastic nature are presented.

## Materials and methods

### Patients

Tumor tissue samples were collected from four patients who had undergone resections for cystic pancreatic tumors between 2004 and 2010 at the Department of Surgery, University of Heidelberg (Heidelberg, Germany). Clinical data were collected from the files of the Department of General Surgery, University of Heidelberg. The study was approved by the ethics committee of the University of Heidelberg (no. 206/2005 and no. 301/2001). Patients provided written informed consent.

### Microscopy and immunohistochemistry

Tumor tissue specimens were formalin-fixed and paraffin-embedded, sectioned (4 μm) and stained with hematoxylin and eosin. Immunohistochemical analyses were performed with a primary polyclonal mouse anti-human antibody directed against trypsin (1:2,000; Qed Bioscience Inc., San Diego, CA, USA), monoclonal mouse anti-human antibodies directed against cytokeratin 7 (1:50; clone OV-TL 12/30; DakoCytomation, Glostrup, Denmark), cytokeratin 18 (1:10; clone DC10; DakoCytomation), synaptophysin (1:2; clone Snp 88; BioGenex, San Ramon, CA, USA), chromogranin A (1:2; clone LK2H10; Linearis Beratungs-GmbH, Wertheim, Germany), Ki-67 (1:100; DakoCytomation), p53 (1:100; clone DO7; DakoCytomation), β-catenin (1:200; clone 14; BD Transduction Laboratories, Lexington, KY, USA) and epidermal growth factor receptor (1:50; clone 31G7; Zymed Laboratories Inc., San Francisco, CA, USA), as well as a polyclonal rabbit anti-human antibody directed against Smad4 (1:50; rabbit polyclonal; Santa Cruz Biotechnology, Inc., Santa Cruz, CA, USA) using the avidin-biotin complex method. If necessary, antigen retrieval in the sections was achieved by microwave pretreatment in citrate buffer (used for trypsin, p53, Smad4 and β-catenin).

### Assessment of clonality and mutation analysis

Genomic and mitochondrial DNA (mtDNA) was isolated from two different areas of the lesions as well as from two or three foci of adjacent normal acinar tissue using microscope (Axioskop 2 plus, Carl Zeiss, Jena, Germany)-assisted manual microdissection as previously described (12). To assess clonality, the highly variable displacement-loop region (nucleotides 16,045 through 650) of the mtDNA was amplified in two fragments using the primers published by Morandi *et al* (13) followed by bidirectional sequencing as previously described. Multiple sequence alignments were performed for each case using the ClustalW software (14), and dendrograms and relative distances were calculated using Jalview (15).

For mutational analyses, exons 1 and 2 of the *K-ras* gene and exon 3 of the *β-catenin* gene were amplified by polymerase chain reaction (PCR), using primers that have been previously described (16,17). Following control of the PCR fragments by agarose gel electrophoresis and purification of the probes (High Pure PCR purification kit; Roche Diagnostics, Mannheim, Germany) all probes were bidirectionally sequenced using an ABIPrism 377 DNA sequencer (Applied Biosystems, Darmstadt, Germany) using the DYEnamic ET Terminator kit (GE Healthcare, Freiburg, Germany).

## Results

### Clinical observations

As summarized in Table I, the two female and two male patients were aged between 25 and 62 years (average, 48.5 years). The patients’ symptoms were non-specific and included weight loss, pain and abdominal discomfort. In one patient, the tumor was identified incidentally during a gynecological check-up. In the patient histories, which are described in detail in Table I, none of the patients presented with previous malignant neoplasms. Furthermore, no pancreas-related diseases were identified with the exception of non-insulin dependent diabetes mellitus in one patient. In addition, all patients were smokers. Preoperative tumor markers (obtained from three patients) showed normal levels of carbohydrate antigen 19-9 and marginally elevated carcinoembryonic antigen levels in two patients. Imaging techniques revealed cystic pancreatic lesions in all patients, however, metastases were not detected.

Depending on the location and size of the lesions, pylorus-preserving total pancreatectomies were performed in three patients, while one patient underwent a pylorus-preserving Whipple procedure in combination with an oophorectomy for serous cystadenofibroma. The postoperative courses of the patients were uneventful.

### Pathological and immunohistochemical observations

**Figure 1:**
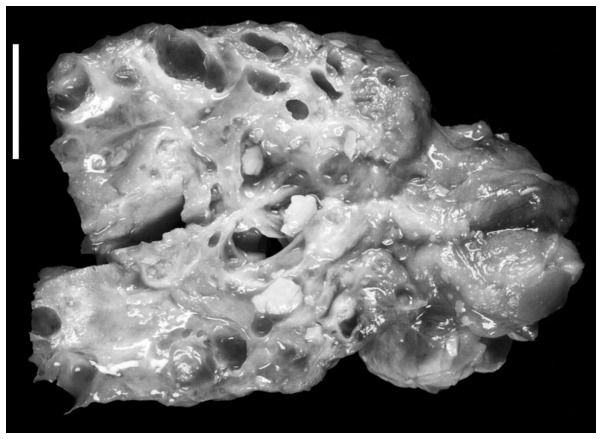
Gross findings in acinar cell cystadenomas. On the cut surface, the tumors exhibit multiple cystic lesions of various sizes that are filled with a partially precipitated, whitish fluid (shown for patient 2; scale bar, 1 cm).

At gross examination, the pancreatic surgical specimens contained multiple cystic spaces that were filled with a partially concentrated, clear to white serous fluid (Fig. 1). The size of the cysts within the multiloculated lesions usually measured only a few millimeters, but reached up to 3 cm in the largest diameter. In addition, the lesions were ill demarcated and lacked a capsule. As summarized in Table I, the lesions affected the entire pancreas in three patients, and were limited to the pancreatic head in one patient.

**Figure 2:**
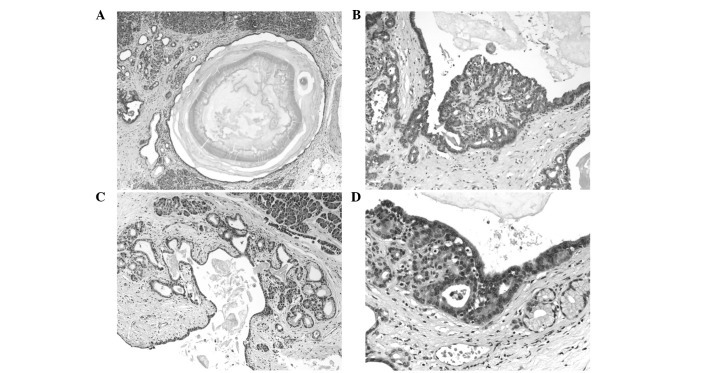
Histological observations in acinar cell cystadenomas. Microscopically, the tumor exhibited multiple cystic lesions of various sizes. (A) The cystic lesions frequently contained serous fluid with abundant lamellated precipitations. Within and adjacent to the cystic lesions, small buds of (B) intraluminal and (C and D) extraluminal acinar cells were observed. (C) With a varying extent, the atypical acinar epithelium exhibited a mucinous transformation (as indicated by the arrow). Magnifications of (A) ×18; (B and C), ×36; and (D), ×72.

Microscopically, the lesions consisted of multiple cysts of varying size that were delineated by one or two layers of acinar cells of mostly cuboidal and occasionally flattened shape (Fig. 2). The cytoplasm was basophilic and, apically, frequently contained eosinophilic granules. In general, the nuclei were located basally and showed an ovoid to oval shape. The majority of cells contained prominent nucleoli, and plurifocal buds and small clusters of atypical acinar cells merged with the cystically arranged acinar cells, occasionally resulting in a rete-like appearance. The cysts were frequently demarcated by a thin layer of connective tissue; however, multifocal small cysts were also identified within otherwise normal appearing pancreatic lobules. Irrespective of their size, the cysts were filled with an eosinophilic fluid with concentrically lamellated protein precipitations. Focally, all lesions exhibited areas of mucinous transformation, the latter being marked in patient 4.

In certain areas, the peritumoral pancreatic tissue showed chronic inflammation and atrophy of the exocrine parenchyma to a varying extent. Furthermore, pancreatic intraepithelial neoplasia (PanIN) 1 lesions were detected in the peritumoral pancreatic tissues of all patients.

**Figure 3:**
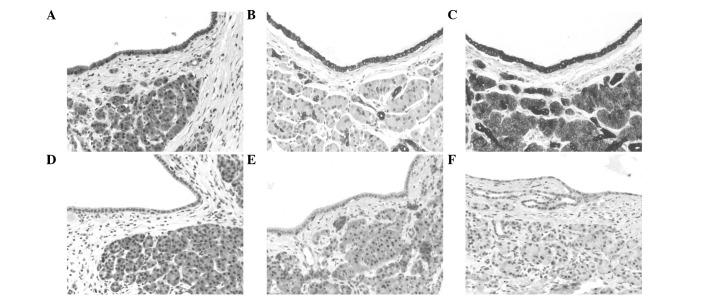
Immunohistochemical observations in acinar cell cystadenomas. The tumor cells revealed a marked cytoplasmic immunopositivity for (A) trypsin, (B) cytokeratin 7 and (C) cytokeratin 18. (D) The proliferative activity, as determined by Ki-67, was <1%. (E) The tumors contained only sparse neuroendocrine cell staining for chromogranin A (shown) and synaptophysin. (F) The tumors did not show nuclear accumulation of p53 (magnification ×72).

Immunohistochemically, the acinar differentiation of the lesions was demonstrated by a strong and diffuse staining for trypsin (Fig. 3). Furthermore, a diffuse and strong coexpression of keratins 7 and 18 was observed. A minor subpopulation of cells lining the cysts (<1%) exhibited immunoreactivity for the neuroendocrine markers chromogranin A and synaptophysin in all cases, and extremely few cells revealed immunopositivity for keratin 20 (patients 2–4). The proliferation rate, as determined by Ki-67 (Mib1), was <1% in all cases.

### Molecular observations

**Figure 4:**
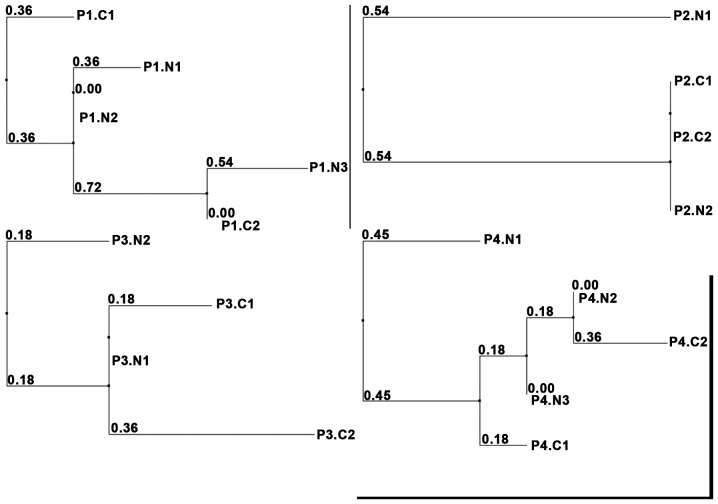
Dendrograms of multiple sequence alignments based on the mtDNA sequencing results in the four cases of acinar cell cystadenomas (patients 1–4). Numbers between branches present relative distances. Cyst epithelia are designated as C1 or C2, and non-involved, surrounding pancreatic tissue as N1–N3. In patients 1, 3 and 4, no evidence for a clonal association between the different areas of the cystic lesions was apparent. The sequence alignment obtained from patient 2 was considered non-informative with regard to clonality, as identical mtDNA sequences were obtained from two positions of the cysts and the adjacent, non-involved pancreatic parenchyma (N2). mtDNA, mitochondrial DNA.

Sequencing analysis of the D-loop region of the mtDNA was successfully performed on two different microdissected areas of the acinar cell cystadenomas in all cases, as well as in two (cases 2–3) or three (cases 1 and 4) adjacent areas of normal tissue. To compare the average number (and type) of mutations between the obtained mtDNA sequences from different foci of the cystic lesions and adjacent normal tissue, a multiple sequence alignment was computed. Dendrograms, including relative distances, are shown in Fig. 4. In case 2, identical mtDNA sequences were obtained in the two DNA preparations of the acinar cell cystadenomas, but also in one of the normal tissue samples, indicative of a close correlation between the cells in all three preparations. However, in all other cases, diverging mtDNA mutations between the different acinar cell cystadenoma foci were observed (eight, three and five mutations in cases 1, 3 and 4, respectively), the average number of mutations between acinar cell cystadenomas and adjacent normal tissue was 4.05 (median, 3; range, 0–11). Thus, comparison of the mtDNA sequences as well as the visualized distribution of the lesions in the calculated dendrograms showed no closer correlation between the different acinar cell cystadenoma foci as compared with the adjacent normal pancreatic (acinar) tissue.

Bidirectional sequencing revealed no *K-ras* mutations in exons 1 and 2 (encompassing codons 12, 13 and 61), and no *β-catenin* mutations were identified in exon 3. Immunohistochemically, the tumors showed significant nuclear accumulation of β-catenin. Furthermore, no nuclear accumulation of the p53 protein was found by immunohistochemistry (Fig. 3), and the tumors revealed a regular expression of Smad4/DPC4.

## Discussion

Currently, the biological nature of pancreatic acinar cell cystadenomas is discussed controversially. It has previously been suggested that these lesions may be non-neoplastic and present cystic transformation of single acini or clusters of acini, possibly due to a cell differentiation failure (11). Arguments in favor of this hypothesis include the observation of the present and previous (3,11) studies that acinar cell cystadenomas often exhibit an intimate admixture of the cystic structures with dilated but otherwise normal appearing acini (3,11). As shown in the current study and a previous study (8), the cysts are lined by frequently heteromorphous cells, consisting of easily identifiable acinar cells, flattened, duct-like cells, as well as mucinously transformed cells. This high plasticity, which is also reflected by an immunhistochemical overlap of acinar and ductal characteristics, may be explained by metaplasia of acinar or centroacinar cells, a process that has been described in the setting of inflammation and carcinogenesis (17,18). On the other hand, a neoplastic nature of acinar cell cystadenomas has been suggested, mainly due to their tumor-like appearance (4,11). The degree of cellular atypia and mitotic activity is generally low and, in the available cases, no progression into higher grades of dysplasia or even malignant transformation has been reported (4–11). In a recent series, mural nodules were observed in two cases. These cellular nodules were exclusively composed of acinar cells, intimately admixed with cysts, and reached sizes of up to several millimetres (8). Recognizing the possibility that a subset of acinar cell cystadenomas may be caused by non-neoplastic proliferations, Khor *et al* (8) interpreted the presence of these mural nodules as evidence for a neoplastic process.

The molecular biology of acinar cell cystadenomas is poorly understood and, to date, no molecular analyses of the cystic lesions have been performed. Using mtDNA sequencing and clustering analyses, the current study demonstrated that at least three of the four cases of the series presented polyclonal lesions, and no evidence for a common clonal origin was observed in any of the cases. This observation indicated that classical acinar cell cystadenomas should be considered reactive or hyperplastic rather than neoplastic. However, in a recent report of 10 cases of acinar cell cystadenomas, two cases were identified in which intramural nodules of epithelial cells with acinar differentiation were present in the cyst walls. In one of these nodules, array-based comparative genomic hybridization identified chromosomal imbalances indicative of a neoplastic process (8). In the context of the results of the current study, this observation may indicate that the formation of intramural nodules of acinar epithelia may present focal transformation into a neoplastic lesion.

Wild-type copies of *β-catenin* and its regular expression as detected by immunohistochemistry in all four cases of the current series suggested that β-catenin is not significant in acinar cell cystadenomas. In acinar cell carcinomas, genetic alterations in the Wnt signaling pathway have been shown in four of 17 investigated cases, including three truncating mutations of the *APC* gene, and one 1-bp missense mutation in codon 41 of exon 3 of the *β-catenin* gene (19). Furthermore, the mutational analyses performed in the present study revealed wild-type copies of *K-ras* in all cases, as well as an intact immunohistochemical expression of Smad4/DPC4. Together with the regular expression of p53, as observed not only in the current study but also in one previous report (11), this clearly discriminates acinar cell cystadenomas from duct-related pancreatic neoplasms, such as PanIN lesions and IPMNs. In these, *K-ras* mutations present frequent and early observations (20–23). Occurring less frequently, alterations of p53 present late events in PanIN and IPMN, which are associated with a high grade of dysplasia (20,23–26) Furthermore, loss of Smad4/DPC4 is an abundant observation in high grade PanIN lesions (25,27). In IPMN, loss of Smad4/DPC4 has been reported to be associated with invasive tumor growth (28), nevertheless, it has been found to present an early change in the progression of these tumors (26). In acinar cell carcinomas, mutations of *K-ras, Smad4/DPC4* and *p53* were not reported to be significant (19,29–33).

Subject to the small number of available cases (4–11), including the present series, females appear to have a higher risk of developing acinar cell cystadenomas than males, as 19 of the 30 patients (63%) were female (Table II). This diverges from acinar cell carcinomas, in which the proportion of female patients has been determined to range between 36.5% (34) and 46.4% (35) in large clinical series. As conveyed from the observations of the current and previous studies, clinical symptoms associated with acinar cell cystadenomas appear to be non-specific and most frequently include abdominal pain and/or discomfort. Notably, two patients of the current series presented with weight loss. From the observations of the current and previously reported cases, definite predisposing diseases or factors do not emerge. Markedly, however, all patients in the current series were cigarette smokers, presenting one of the few well-established risk factors for pancreatic ductal adenocarcinoma (36). Nevertheless, no respective information has been provided in the previous reports and, due to the rareness of the tumors, it appears questionable that future epidemiological studies are likely to succeed in identifying putative predisposing factors for acinar cell cystadenoma.

In conclusion, the current study provides molecular evidence that acinar cell cystadenomas not containing mural nodules present non-neoplastic lesions. In addition, genetic alterations typically found in duct-related pancreatic neoplasias were not shown to be involved in acinar cell cystadenomas. Whether rare mural nodules present a focal neoplastic transformation on the basis of acinar cell cystadenoma remains to be clarified; however, it must be considered to replace the term acinar cell cystadenoma by cystic acinar transformation, as previously suggested (37).

## Figures and Tables

**Table I:** Clinical and pathological observations in four acinar cell cystadenomas.

Variables	Patient 1	Patient 2	Patient 3	Patient 4
Tumor location	Entire pancreas	Entire pancreas	Head	Entire pancreas
Gender/age, years	M/25	F/46	F/62	M/61
Symptoms	Weight loss and radiating pain	Abdominal discomfort	Incidental finding	Weight loss
Surgery	PP total pancreatectomy	PP total pancreatectomy	PP Whipple and bilateral oophorectomy	PP total pancreatectomy
History	Tuberculosis in child age and cannabis abuse	NIDDM, goiter and secondary hyperpara-thyreoidism	Goiter, COPD, hysterectomy, fatty liver and depression	COPD, coronary disease and status post myocardial infarction
Smoker	Yes (five py)	Yes (four per day)	Yes (45 py)	Yes (40 py)
Tumor markers (preoperative)	CA19-9, <1 kU/l and CEA, 6 μg/l	Not determined	CA19-9, 32.3 and CEA, 5.5	CA19-9, 4.2 and CEA, 0.8

M, male; F, female; PP, pylorus preserving; py, pack-years; NIDDM, non-insulin dependent diabetes mellitus; COPD, chronic obstructive pulmonary disease; CA19-9, carbohydrate antigen 19-9; CEA, carcinoembryonic antigen.

**Table II:** Summary of clinical and pathological observations of 26 previously reported cases of acinar cell cystadenoma.

Case	Gender/age, years	Tumor size, cm	Macroscopy	Location	Symptoms	Other disease/remarks	Ref.
1	F/33	10	UL	Head	Abdominal pain	None	11
2	F/46	4 and 10	Bifocal UL	Head-tail	Abdominal pain	None	11
3	F/16	7.5	ML	Head	Abdominal pain	None	11
4	F/44	0.1–1.5	Multifocal UL	Diffuse	Polyarthralgia	DM and sarcoidosis	11
5	F/47	0.5–2-5	UL	Head-tail	Abdominal pain	Rheumatoid arthriris	11
6	F/39	4	ML	Head	Abdominal pain	n.i.	11
7	F/49	0.5	UL	Tail	n.i.	Insulinoma	11
8	M/57	0.5	UL	Tail	Abdominal discomfort	Endocrine tumor	11
9	M/66	0.2	UL	Head	n.i.	Intraductal papillary adenoma	11
10	M/61	0.2	UL	Head	Jaundice	Bile duct papillary hyperplasia and intraductal papillary adenoma	11
11	F/58	9	ML	Body-tail	Incidental finding	Myocardial infarction and longstanding DM	4
12	F/40	4	ML	Head	Acute pancreatitis	n.i.	6
13	M/52	5	UL	Body	Abdominal pain and incidental finding at follow-up	Pulmonary adeno-carcinoma	5
14	M/9	11.7	ML	Pancreas	Incidental finding	Acute appendicitis; biopsy only	9
15	M/52	5	Multiple UL	Head-body	Incidental finding	Renal cell carcinoma	7
16	F/55	10	ML	Retro-peritoneum	Abdominal pain	None	10
17	F/42	≥2	Multifocal ML	Head-body	Intermittent discomfort left flank	n.i.	8
18	F/23	6	ML	Head	Epigastric pain	n.i.	8
19	F/31	7.5	ML	Pancreas with peripancreatic extension	Left flank pain	n.i.	8
20	M/65	6.9	ML	Head	Abdominal pain	n.i.	8
21	F/25	2.9	UL	Body	Chronic abdominal pain	n.i.	8
22	M/68	3.5	UL	Tail	Incidental finding at follow-up	Renal cell carcinoma	8
23	F/71	5.1	ML	Head/neck	Intermittent discomfort in epigastrium and right upper quadrant	n.i.	8
24	M/33	n.i.	ML	n.i.	n.i.	n.i.	8
25	F/67	5	Multifocal UL	Head	Incidental finding	n.i.	8
26	F/59	3.2	ML	Head/neck	Incidental finding	n.i.	8

F, female, M, male; n.i., not indicated; UL, unilocular, ML, multilocular; DM, diabetes mellitus.
